# The paradoxes of love in the Spanish family: a sociological approach

**DOI:** 10.12688/f1000research.51358.1

**Published:** 2021-07-21

**Authors:** Juan Antonio Roche Cárcel

**Affiliations:** 1 Department of Sociology I, University of Alicante, Alicante, E-03080, Spain

**Keywords:** Spanish family, society of individualization, confluent love, romantic love, eclipse of family, typology of Spanish families, Family relationship models

## Abstract

This article defines the Spanish family in the context of the “Mediterranean model” and the “individualization society”. The former is characterised by strong social interrelationships between family members and their emotional ties, while the latter is defined by the separateness of citizens and by institutionalising the basis of society in individuals rather than in the family. The work also describes how modern forms of love, both romantic and confluent, are institutionalized in this society, discussing if they coexist or not, how they exist, and which is the dominant form. Finally, it analyzes the degree of strength or fragility of the family institution and the affective relationships that sustain it.

The work concludes that the Spanish family is balancing between the strong resistance to disappear as an institution and its eclipse, crisis, or complete end. This is because, although the Spanish family still retains a large part of its former functions, at the same time as divorce is on the increase and family members are decreasing, it is increasingly ephemeral and a plurality of family forms have emerged that have broken with the traditional dominant model of lifelong romantic marriage. Moreover, the Spanish family is also among the “familist” model and the individual, while the way of loving fluctuates between the traditional patriarchal and the democratic, individual, and communitary. Thus, the thesis I propose qualifies and questions the majority of theoretical works on love and the Spanish family, which argue that the family is inscribed in the “Mediterranean model”. As will be seen, the romantic relationships that have been institutionalised in the Spanish family are more paradoxical, insofar as they are still inscribed in that model, but they are rapidly approaching those of Northern Europe.

## Introduction

Sociology is concerned with love because of its enormous capacity to build bonds, ties, and social interrelations, as well as constituting a fundamental component of the family and the conjugal couple. The discipline has also classified the types of love characteristic of modernity. Thus, based precisely on some of the sociological, theoretical, and empirical work that has analyzed the interrelationship between the Spanish family and love, the objectives of this article are the following:

1) To contextualize the Spanish family with regard to the society of individualization and define the basic features that characterize it.
2) To reveal how modern forms of love, and above all the romantic and confluent forms, are institutionalized, formalized, or “familiarized” in Spanish society.
3) To discuss whether different types of love coexist and how they do so, to reveal which is the primary and which is the secondary form.
4) To provide evidence of the degree of strength or fragility of the family institution and the emotional relationships that support it.

To achieve these ends, I have structured this article in sections dedicated to the sociological treatment of love and modern types of love, including the five basic aspects of the Spanish family and the coexistence of these modern types of love, namely romantic and confluent. Finally, I will conclude that love in Spain is characterized by its individuality and its minimalism: that the five basic characteristics of the family express a strong tension between the resistance to disappear and its tendency to eclipse, crisis or end and the two types of modern love - the romantic and the confluent love - coexist with other models.

## The sociological treatment of love and the two modern types of love: romantic and confluent

Love constitutes one of the fundamental themes of human existence, understood in an individual and social way. It is, in fact, a multifaceted, multidimensional, and polysemantic issue that requires an inter- and transdisciplinary approach. As far as sociology is concerned, since its origins with Weber in a form of modern rationalism ( Weber, 1975, 1979, 1987, 1997; Bellah, 2005, p.130-131), it neglected the general study of the field of emotions and, in particular, of love. Thus, although it certainly has classical antecedents that referred to this emotion, it was always considered a badly seen subject ( Jónasdóttir, 2014). Before the publication of N. Luhmann’s book, *El amor pasión,* 2008 (originally published in 1985), sociology did not have a monograph exclusively dedicated to this essential and complex issue ( García Andrade, 2015, p.56).

Since then it is true that the road traveled in sociological research around the subject of love has been long and fruitful, to the point that numerous theoretical and empirical articles and important books dedicated to love have appeared; finally, love has assumed a central role in the discipline. However, this does not mean that a canonical paradigm has been established nor that there has been a homogeneous approach, but rather, on the contrary, that plurality, divergence, and contradictions are the norm ( García Andrade, 2015, p.37).

This has not been an obstacle since, throughout the history of the subject, sociologists have referred to the capacity of love to produce bonds, ties, and social interrelations ( García Andrade, 2015, p.56). For example, N. Elias in *La sociedad Cortesana,* 1996, highlights love’s axial role in social bonding, which is affective, has a strong intensity and builds a sphere of meaning ( Sabido and García, 2015, pp.22, 38).

I am particularly interested here in sociologists who have analyzed love as a fundamental component of the family and the conjugal couple (Cicchelli-Pugeautl, 1999, p.58). Thus, for Luhmann, the family is ordered based on a particular code of symbols, one being love (2008, pp.24, 203), and since the 18th century the unity between love and marriage has been observed as a principle for the perfect fulfillment of the human being. François de Singly, for his part, has drawn attention to the central role of the family in the process of revealing the self, of building individualized identity (2016, p.28) and, according to Ulrich and Elizabeth Beck, the family has moved from being a working community to one of feeling ( Beck and Beck-Gersheim, 2008, pp.19-76). For the philosopher Alain Baidou, the family builds the state of love ( Badiou, 2011, p.72) and according to Bruckner and Finkielkraut, behind this state of love there is the desire of the institution to conjure up chance ( Bruckner and Finkielkraut, 2001, p.327). While other social thinkers have emphasized the transformations that the modern family is undergoing, as is the case with Marx, who prophetically anticipated that the family, once the process that will lead teleologically to communist society is concluded (in this he was wrong), will be formed by a couple united by pure erotic-individual love ( González, 2009, p.513). Durkheim, for his part, understands that due to the ‘law of progressive contraction’, the family will diminish in size as the division of labor in society grows ( Segalen, 1992, p.28). Although the Frankfurt School does not defend the disappearance of the family, it considers that the bourgeoisie has come to an end, which has led to the emergence of other family forms, basically expressive and egalitarian ( González, 2009, p.524).

Therefore, as suported in the claims above, the family constitutes a great expressive and equalizing force and builds on the state of love. Conversely, love is a fundamental component of the family and the conjugal couple and love then becomes its basis: love is family and family is love.

As for the types or styles of love, there have been various classifications made by social scientists. One of them proposes that, in summary, today there are two models of affective-sexual relationships - traditional and alternative - but they are paradigms of “ideal type” behavior ( Gómez, 2008, pp.63 ff.). Another of the most accepted categorisations in sociology is that proposed by Anthony Giddens in *The Transformation of Intimacy: Sexuality, Love and Eroticism in Modern Societies,* where he highlights the two variants of modern love: the “romantic” and the “confluent” (2006, pp.43-63).

The first of these emerged at the end of the 17 ^th^ century, and was defined by sociology as the First Modernity, from 1890 to 1973, after which the Second Modernity then begins ( Bell, 1992, p.117). This is a legacy of the “courtly love” of the feudal system ( Elias, 1994, p.325 ff.) and the “passionate love” characteristic of the absolutist aristocracy, linked in turn to the Platonic “eros” and the Christian “agape”. However, as will be discussed, this romantic form of love is still very strong today, particularly in Spain, where it relates to the majority of the population because the current patriarchal and capitalist system effectively reproduces it through the norms, beliefs, models, customs, myths, traditions, morals and ethics of the culture of which Spanish citizens are a part ( Herrera, 2018, p.10). Moreso, literature, cinema, and television soap operas represent it, revive it, and re-actualize it ( Elias and Dunning, 1996, pp.94-95), in the same way that the sacred rites of the church keep Catholic religiosity alive. It is not surprising that this type of love contains a literary component, even melodramatic, linked more to the mental than to the carnal, more to passion than to sex, and that, consequently, is an ideal, which usually is never completely fulfilled. Without forgetting that it constitutes a pure relationship that restructures intimacy and is characterized by the fusion of the couple, in such a way that the other ceases to represent otherness. The spouses renounce being individual beings and, simultaneously, separate themselves from their broad social context, for example, from institutions or the friendship group, and even from everyday life. In this way, romantic love creates a shared, relational, and non-individual story that, like fiction, flees or duplicates the world.

However, paradoxically romantic love is interrelated with the idea of the free choice of the couple and with the consequent conception of freedom and individual self-fulfillment. This vision reaches its maximum ardor (i.e. this love is very intense) in women, who believe that they obtain maximum happiness through marriage, the ultimate sustenance of romantic love. The latter gives them independence and identity, while at the same time, with the affections and emotional ties it generates, it almost reaches the sublime, an almost sacred feeling. In contrast, in men, this idea of freedom and self-realization is manifested through success and professional development. That is, while the husband, with his external function in the family, mainly out at work, and the instrumental role that this work gives rise to does not concern himself with expressive roles, which instead are adopted by the wife. Hence, romantic love is feminized, not in vain, it seems - as Giddens (2006, p.43 ff.) points out - that it has been an invention of women, like motherhood and childhood, from the changes produced in the home itself, to the extent that it is transformed from a space of production and reproduction to another in which affection dominates. Women have indeed empowered themselves in the home, have taken control of its administrative and economic management, and have adopted a greater weight in the education of minors. These facts have led to the weakening of the traditional patriarchal functions and, ambivalently, to the parallel reinforcement by them of the reproduction of the patriarchal and capitalist system.

Finally, romantic love implies a continuous search, a work ( Gómez, 2008, p.49) and in Erich Fromm’s (2007) vision an artistic expression, a day-to-day work, a cultivation ( Cruz, 2010, p.187), and a total dedication which constitutes a process that has no purpose. However, it is projected into the future, insofar as it is a love that seeks to last a lifetime and that, if it could, would even border on transcendence and immortality.

Confluent or “liquid love”, characteristic of the Second Modernity, is defined sociologically with at least five characteristics ( Giddens, 2006; Beck and Beck-Gersheim, 2008; Costa, 2006, pp.775-778; Bauman, 2007, pp.32 ff.; Illouz, 2009, p.205):

1. It pursues the free and not closed construction of the individual members of the couple, not the fusion - as romantic love does - because what it seeks is to open up to the other and, therefore, to understand him or her. It is defined, therefore, by individualism ( Simmel, 1986, p.96; Elias, 1999, p.49; García Andrade, 2015, p.55), by the self-confirmation of the *self* or by the self-realization of the individual (Berger and Kellmer, 1993, p.226). In short, it is defined by the defense of individual autonomy (Ayuso, 2015, p.90).
2. It presupposes the equality of its members and requires that, in the interrelationships between them, there is reciprocity or “intrahuman interpenetration” ( Luhmann, 2008, pp.233 ff.). For this reason, it is a more democratic, more “negotiating” love than the more aristocratic and authoritarian romantic version.
3. It builds lighter personal ties and, consequently, fragile, “liquid” ( Bauman, 2007), contingent, ephemeral, and uncertain relationships. Thus, although it may exist today, it does not necessarily do so tomorrow and hence is intense and active, without this meaning that it lasts forever; indeed, it is an instantaneous love. As undesired effects of this inconsistency, there is an increase in separations and divorces, while the loving subject becomes a disposable consumer object, i.e. a commodity determined by the frenetic rhythms of the capitalist system.
4. It is a risky love, in that it assumes a whole series of dangers and insecurities that put its own survival at stake, since it escapes from the protection and control of the family and since those in love invest in feelings, in economic, symbolic and value capital, following the economic laws of supply and demand. However, there is neither profit nor happiness guaranteed - especially in the case of women ( Jónasdóttir, 1995, p.314).
5. It highlights the *ars erotica,* sexuality, although this decreases over time, with its short duration. However, it is not exclusively linked, like romantic love, to heterosexuality.

## The Spanish family and the individualistic model of Northern Europe

Next, I will discuss how the two modern forms of love - romantic and confluent - are institutionalized, formalized, or “familiarized” in Spanish society. Specifically, I will first contextualize these two types of love in the unique Spanish family model and its family structure. I will then explore if and how the two forms of love coexist, to reveal which is the primary and which is the secondary form.

It has been pointed out that, in Europe, there are two different family care models between the North and the South ( Holdsworth, 2000, p.201). In the first, the “Bismarkian”, there is explicit government support, through specific fiscal and social policies, for the role of the family as a welfare and care provider, mainly through women’s unpaid care work. In the second, the “Mediterranean” model, families carry the burden, replacing the absense of public or state intervention. The existing literature so far has defined the Spanish model as Mediterranean or “familist”, due to the primacy of the family in the protection of dependents. However, recently the Spanish model, in general, and the family principle in particular, is being revised ( León and Migliavacca, 2013, pp.25, 35). It has even been pointed out that the Spanish welfare state based on familism may be in crisis. This is the result of the fact that the Spanish family has undergone numerous changes linked to the transformations of society, principally with the economic and industrial development initiated in the 1960s and with the transition and consolidation of democracy. Specifically, there have been numerous external economic, social, cultural, and political variations that have had an impact on this institution. Next to these, there are also internal demographic metamorphoses, in attitudes and behaviors, in the rights and status of family members, in couple relationships and those of parents and children, in marital harmony and couple symmetry (Iglesias, 1990, p.236; Carrasco, 1997, p.90; Del Campo and Rodríguez-Brioso, 2002, p.104).

These profound economic, cultural, and social changes in Spanish families have influenced the transformation of the dynamics of family solidarity (Cais and Folguera, 2013, p.573). Indeed,

[f]amily exchanges may be becoming less driven by duty and more open to individual variation, so that personal affection and attachment may be increasingly important for family cohesion and intergenerational ties. Normative obligations remain in place but may be increasingly being modified by affection and choice so that relationships within the family are being transformed ( Lowenstein and Daatland, 2006, p.219).

Consequently, while intergenerational relationships and solidarity within the family still enjoy social prestige and while a strong and ingrained social custom survives, people’s preferences for family care are less driven by duty and more dependent on affection and personal attachment. As a result, Spaniards no longer feel an inescapable moral duty towards their dependent parents, and they do not spend as much time caring for their relatives as they once did. On the contrary, normative beliefs have become more flexible and have adapted to new social realities such as gender equality and the increase in women’s participation in paid work (Cais and Folguera, 2013, pp.558-573).

From the mid-1990s to the mid-2000s there has been strong growth in female employment in the country from 20% in 1992 to 52% in 2010, with the female employment rate (between 15 to 64 years old) increasing from 46% to 64% between 2004 and 2008. Besides this, there has also been a notable increase in their level of education, especially among the younger generation, from 8% in 1987 (plus another 16% of those in primary school) to 30% in 2007, without forgetting that those reaching tertiary education increased from 6% to 16% ( Salido, 2011, p.190; León and Migliavacca, 2013, pp.29-31).

Thus, in 20 years Spain has seen an increase of 25% in female workers and has gone from being the country with the lowest number of women in the workforce in the European Union to having a volume of female labor comparable to other countries such as France, Germany or the United Kingdom. As a result, the number of women who are exclusively “employed” in the home and who form the basis of the family has halved over the last two decades. Thus, if in 1988, 42% of those women were dedicated only to household tasks, in 2008 this figure has fallen to 23% (Cais and Folguera, 2013, pp.561-562; León and Migliavacca, 2013, pp.29-30).

Consequently, since the 1990s, and decisively facilitated by mass migration - a key element in the configuration of care systems in Southern Europe - a rapid process of commoditization of care work has also taken place, although still within the confines of the home. This is also in line with common trends in other European countries ( León and Migliavacca, 2013, p.38).

Likewise, the Spanish family model has converged with that of the European family due to its immersion in the individualization society ( Meil, 2003, p.2 ff.; Meil, 2004, p.421). Indeed, there has been an increase in individualism and in the ethics of personal self-fulfillment ( Flaquer, 1991, p.70), especially in women, who have moved from a life model oriented towards family service to one in which they assert their right to have their own professional career (Cais and Folguera, 2013, p.561). This has led to the predominance of individual interests and rights over those of the family institution (Mora, 2012, p.102). This means that the variety of lifestyles is wider (Cais and Folguera, 2013, p.561) and that what is really at stake is the place of the individual within society ( Del Campo, 2004, p.452). In other words, the lives of citizens are no longer planned by society, which has forced them to build their own lives, with all the risks that this new situation entails. Therefore, from having a conventional biography, more robust and built over time, they have come to have a more flexible one, something which denotes a greater fragility in relationships and the loss of certainty ( Beck, 1988, p.217).

As a result, over the last three decades, the ability of the family in Spain to care for its dependent members, especially the disabled and elderly, has been affected by two transformations. The first is that changes in family structure - particularly the increased participation of women in the labor market - have been accompanied by the changes experienced in family dynamics. This is because people’s preferences about how to care for dependents are now less motivated by moral duty and more by a sense of responsibility that can be addressed by different means, as well as by affection and personal attachment (Cais and Folguera, 2013, pp.560-561). Hence, it has been written that the inherited social rules that have defined the structure and roles of family members in the past have become obsolete and that the figure of the woman dedicated exclusively to domestic work and the care of children, parents, and husband is dying (Mari-Klose, 2006). It is not surprising that the merging of employment and education levels among the younger cohorts of women during the 1990s and the first half of the 2000s, which is much more pronounced in the Spanish case than in other European countries, has been interpreted as a move away from the “southern male breadwinner” model to the northern adult worker model; that is, Spain has moved away from “southern” norms and towards “northern” ones. In a similar vein, it has also been argued that Spain has made the transition from a “male breadwinner model” to a more diversified pattern of family formation concerning women’s participation in the labor market. It is further argued that this male ideal of a breadwinner has long since disappeared and that no one seems interested in regaining it ( León and Migliavacca, 2013, pp.25-36).

However, the process is not as linear as it seems, as it is much more nuanced and complex. First of all, despite the variations in Spain, as all the specialists insist are happening, the family continues to be one of the “friendliest” institutions in Spanish society and is shown to be the most highly valued aspect of life, as the degree of satisfaction with family life and marriage is high. In fact, it is the most important institution in the lives of Spaniards, insofar as 90% of them declare themselves to be fairly or very satisfied with their family life (Sánchez and Bote, 2008, pp.201, 204). Hence, living together as a family is still the most widespread living arrangement, with approximately 86.5 % of households being family-based, the highest rate in Europe (Del Campo and Rodríguez, 2002, p.108). Furthermore, the population’s complacency with the functioning of the couple’s relationship is quite considerable; conflict in marriage is relatively low, marital harmony is desirable, marital break-ups have not reached the European level (Del Campo and Rodríguez, 2002, pp.103-142) and marriage remains the step which comes before procreation ( Delgado, 1993, p.126). This explains why the Spanish family continues to be determined by the intensity of relationships ( Sarrible, 1995, p.48); by a type of interpersonal relation characterized by sociability, proximity, physical affection, and intergenerational family structures ( Lavell *et al.*, 2020, pp.6-8). Likewise the Spanish family is determined by an inherited cultural dimension, based on family solidarity and on the structure of kinship, in which societal norms continue to be important ( Holdsworth, 2000, p.201; Moreno, 2001, pp.1-5).

Also, Spain, together with Greece and Portugal, is the European country where the percentage of people over 65 living in single-parent households is lowest: less than 20% in 2001. Moreover, the percentage of people over 65 living with their children is also higher in Spain (17%), Ireland (15%), Greece (15%), Italy (14%) and Portugal (12%) than in countries such as Germany (1%), Denmark (0.3%), France (5%) or the United Kingdom (6%). It can also be said that families continue to care for people in need. In 2008, for example, 55% of people in need of care had this provided by their families, mainly by children and, secondarily, by spouses (Cais and Folguera, 2013, p.562).

Therefore, in Spain the family continues to be the source of the most support and care for the elderly, while public services are so scarce to the extent that some families are not familiar with them. Actually, the care expectations of elderly dependents are focused on the family and not on social services (Cais and Folguera, 2013, pp.562-564; León and Migliavacca, 2013, p.37). Finally, the 2008 crisis brought a clear decline in female employment, indicating that the level of change developed over the last two decades towards a model of adult worker has encountered a strong obstacle ( León and Migliavacca, 2013, pp.32-35). We still do not know how this situation will end, particularly after the coronavirus crisis.

In sum, this transformation of the state of the family is likely to manifest not as an unequivocal trend towards individualization, but rather a reformulation of the concept of family in terms of roles, functions, and face-to-face relations with other institutions (Daly and Scheiwe, 2010 in Daly, 2011, p.18).

## Five basic structural aspects of the Spanish family

Within this complex general context, the structure of the Spanish family is characterized by at least five basic aspects: its progressive reduction and de-institutionalization; its heterogeneity; its privatization; its negotiating capacity; and the plurality of its forms ( Meil, 1998, p.188; Meil, 2006, pp.11-38; Del Campo, 2004, pp.456 ff; Ayuso, 2015, p.76). Numerous works, both theoretical and empirical, confirm this basic mapping of the Spanish family and simultaneously deepen and nuance its structure while making it possible to reveal the type of love that prevails. I will now discuss these two concepts in further detail.

The Spanish family is becoming increasingly smaller. In 1970, the average size of a Spanish family was 3.81 people, in 2000 it was around 3.7, while in 2017, the Short-term Fertility Indicator is 1.31 children per family ( Eurostat, in Del Campo and Rodríguez-Brioso, 2002, p.105). This means that, on average, the Spanish family has only one child, thus being made up of the father, the mother, and a child, and therefore, the population is not being renewed and is instead decreasing. However, this decrease in the number of members per family unit reflects the slow decline of the nuclear family and its replacement by new forms of cohabitation. Indeed, in addition to the traditional family, which in Spain could be made up of four children or more, other family forms emerge, e.g. families with one or two children or those with no descendants: in 2000, 19% of couples had no children ( Del Campo and Rodríguez-Brioso, 2002, p.110). In this sense the decline experienced from 1998 onwards is the result of the increase in single-parent and single-child families. Therefore, the family in Spain is gradually becoming ‘minimal’, reduced to its minuscule expression, a process which seems to have reached its peak.

In any case, in the end this reduction is an exponent of the contemporary “eclipse” of the Spanish family, or its deconstruction. This is motivated by the passage from family to personal autonomy, by the diversification of types, by the evolution of marriage models, by the conversion of marriage into an individualistic one, with a life phase rather than a life project, and by dismantling or deinstitutionalizing the nuclear family ( Flaquer, 1991, pp.69, 72). In fact, the latter has gone from being a public to a private institution, from closed to open, and from the single model to take a variety of forms ( Del Campo, 2004, pp.454-8), similar to what has happened in Northern Europe ( Meil, 1998, p.188). Together with this, the family has also suffered a loss of centrality in today’s society, since it has ceased to be the primordial institution and has become just another one among others, with few functions of its own: among them, fundamentally, that of being the seat of affection and the socializing agent of individuals ( Del Campo, 2004, p.453).

In any case, this phenomenon affects the intensity and quality of the relations between its members and, particularly, between the spouses, who are, of course, focused on the education of a child, although they also have enough energy left – which is projected as love - to devote to themselves.

The Spanish family is also increasingly heterogeneous, mainly due to changes in their models and their formation processes ( Delgado, 1993, p.123), in the formalization of couples (Iglesias, 2003, p.15), in gender roles and in the affective forms of love. This is expressed through four factors: the pluralization of the ways of living together, the greater heterogeneity in the participation of paid work, the superior heterogeneity in domestic tasks and in the care of the children, and the increasingly heterogeneous work roles inside and outside the home. Indeed, plurality is the most dominant form of cohabitation between couples. Thus, in Spain there are de facto couples, extended families, functional or flexible, nuclear or nuclear-marital, single parent, homoparental, bicultural or multicultural (transnational), simple mixed, complex mixed, single parent, and simultaneous or reconstituted ( Rondón, 2011, pp.82-5). In this pluralization of ways of entering into, remaining in, and leaving family life, however, it should be pointed out that marriage, as a way of living a common life, has been losing its binding force - the marriage rate was 7.50 in 1950 and decreased to 5.12 in 2001 - and even appears to be void of ritual significance ( Del Campo and Rodríguez-Brioso, 2002, pp.117-8, 161). However this does not mean that its social acceptance has deteriorated ( Meil, 2004, p.430). In the same vein the number of nuclear Spanish families has seen a reduction: in 1968, 66% of families were comprised in the form of a nuclear family, in 2001 the percentage fell to 54%, in 2004 the number fell to 50% and in 2018 it reached a low of 33.9% according to the Spanish Office for National Statistics ( INE). Despite the declining numbers, this family form is still the majority, although it is decreasing and approaching European levels -in 2011, the percentage of European nuclear families was 33.2% (Eurostat). The number of unmarried couples has also been increasing in recent years, approaching 12% in 1998 and 15.7% in 2019 ( INE), although this may be associated with the absence of a family project, the lack of professional stability, and the fact that it is harder to own a home together. If this were proven to be the case, it would constitute a transitory form pending its regularization as a new form of nuclear family ( Meil, 2004, pp.435, 446). As for single-parent families, those in which the responsibility lies with the mother have increased by 41%, whilst single-parent families under the responsibility of the father have increased by 60%: in total, in 2018 they accounted for 10.1% of families ( INE). For their part, one-person families – who made up 25.5% of the total number of families in 2018 ( INE) have grown by a high percentage ( INE) ^1^, especially in large cities where more people live alone ^2^, especially older women ( INE) ^3^. Households that consist of only an adult couple without children make up 21.1% of the total and those households in which the family nucleus takes in people from outside the home constituted 4.3% of the total in 2018 ( INE, 2018).

With regards to the participation of fathers in paid work, the incorporation of women into the labor market - particularly in sectors that have not traditionally been female friendly - increases the heterogeneity of family members present in the labor market, which until then had been composed only of men. Thus, as suggested by Alamillos Guardiola, in half of Spanish households, both spouses are working, but half of the women are unemployed, as is the case in all the Mediterranean countries, which mainly affects those women with less education. In contrast, urban and middle-class women have more work than those who live rurally and that of lower-class women. On the other hand, there are more part-time working mothers than fathers, due to the incompatibility between motherhood and their professional career ( Alamillos Guardiola, 2016, p.219).

With regards to heterogeneity in domestic work and childcare, despite being in a country with one of the highest equality awareness rates – 83% of citizens agree with it – it is not put into practice ( Meil, 2003, pp.1-16; Meil, 2006, p.11 ff.). Domestic work is still unevenly distributed (Ajenjo and García, 2014, p.467), with women spending 38 hours a week doing domestic chores and men barely reaching 23 hours ( INE, 2016). Regarding childcare, the distribution of hours is more equal, as both parents dedicated 16 hours per week in 2016 (source INE). Therefore in general, women continue to devote more hours to domestic chores, although men have been able to devote more hours to childcare. It is precisely the education of children that is the task most accepted by parents and the best distributed of all, although it does not include all responsibilities such as going to school to talk to teachers (Meil, 1997, p.3). Moreover, another interesting observation is that in 40% of families where both spouses work outside the home, domestic tasks are shared, although not entirely equally. This generates conflicts within the couple, although the females do not demand the domestic participation of the males in order to prevent creating further conflicts because this would be considered as questionning the established gender roles so that they end up doing more domestic chores Finally, concerning the heterogeneity of paid and unpaid work, in short, women continue to spend the most time working at home, which means that those who work both domestically inside and professionally outside the home, in a large number of cases resort to taking anxiolytics. Indeed, various surveys indicate that more than 50% of Spanish women take anxiolytics and that one of the most important causes leading them to do so are gender asymmetries, work-related asymmetries between women and men, and the fact that women are more likely to take anxiolytics than men (Martínez, 2003, p.253 ff.).

The Spanish family has also privatized daily life, behavior, and morals ( Flaquer, 1991, pp.68-71), as a result of the questioning of social norms and the relaxation of social control, which has created greater freedom and growing egalitarianism among its members ( Rondón, 2011, p.87; Ocón, 2006, p.172). Within this privatization, sexuality stands out, a private expression and personal fulfillment that is not conditioned but rather disconnected from reproduction and marriage ( Delgado, 1993, p.124; Del Campo, 2004, p.459; Sánchez and Bote, 2008, p.209). In this sense, there is a tendency in families to differentiate between love and sexual fidelity ( Meil, 2004, pp.424-6).

All this has meant, on the one hand, that despite significant and profound progress in the cultural, economic, and political fields, Spain has retained a very traditional family system in the context of a democratic society ( Flaquer, 2002, pp.84-85). But, at the same time this traditional family is also becoming more flexible (Mora, 2012, p.103) and is even being transformed from patriarchal or matriarchal authoritarian to democratic which entails a more peaceful and negotiating character (Ocón, 2006, p.172; Meil, 1998, p.193; Rodríguez-Brioso and del Campo, 2002, pp.133, 169). This transformation is due, in turn, to a variation in the relations between spouses and in the paternal-affiliation relations, moved by the increasingly emotional and affective interrelations between family members, as has been seen with the care of the elderly. The family of the 1950s was based on discipline, on family control rooted in an ironclad and authoritarian upbringing, as well as on the imposition of rules and the “domestication of beasts”. In contrast, today’s family is less rigid and more emotional and between the members of the couple and their children, the imposed rules have been replaced by negotiation and shared decisions taken jointly:this was the case for 64% of families in 1995 ( Rondón, 2011, p.88).

In short, these five major transformations of the Spanish family have broken with the traditional system, which could be summarized in two major transformations: interculturality and gender equality ( Rondón, 2011, p.88). However, for all that has been said both with regard to the family model of care and the structure in which it is implemented, the deepening of the social reality of our families is not without ambivalence or, as L. Flaquer, one of the most important experts in the sociology of the Spanish family, points out, it is “paradoxical”: a family paradox (2002, pp.84-85). This explains how, although the family has reduced its institutional charactertistics, it has retained its importance and significance on a factual and ideological level ( Alberdi, 2005). On the other hand, its de-institutionalization, minimalism, and fracture in a multitude of forms may make us think that the family, although it refuses to disappear, does not seem to have much of a future in Spain. ( Del Campo, 2004, p.451).

## The coexistence of the two types of love - romantic and confluent - with other models in the Spanish family

This resistance and fragility of the Spanish family is not, however, the only ambivalence. As will be discussed, at least two other ambivalences are evident: love put into practice is ideal or real and the ideological system it expresses is, respectively, patriarchal, or democratic. For this reason, the analysis of the formalization, institutionalization, and “familiarization” of love is twofold. This is because permanence and change go hand in hand; the replacement of the old alliance between families by love as the constitutive foundation of marriage, has given rise to a dynamic in the social structure, towards an increasingly plural, negotiating, heterogeneous, privatized, less institutionalized and more minimalist family, as has just been described in the previous section. Thus, simultaneously, there has been both the preservation of the authoritarian patriarchal system and the opening up to a more democratic one, characterized by a mechanism of upward social mobility and by the weakening of the stability of the family institution itself ( Meil, 1998, p.191). This explains why, on the one hand, the ideological structure of the Spanish patriarchal family has changed little or less rapidly in practice (García de León, 2009, pp.209 ff.; Castells and Subirats, 2007, pp.22-5). On the other hand, even though family culture is inserted into the Second Modernity, the same has not happened with affective behaviors ( Meil, 2004, p.432), more characteristic of the First Modernity.

I delimit the scope of this contradiction by analyzing several empirical investigations and contrasting them with the theoretical statements made, which I have echoed in the characterization of the care model and the Spanish family structure. It has been stated that the Spanish family has been transformed in the Second Modernity, since, in general terms, the first family modernization was characterized by permanence, by the conditioned choice of marriage, by cultural uniformity and homogeneity. On the contrary, the second modernization is imperishable, develops a personality, freely chooses the couple, and its members adopt a more egalitarian and shared responsibility ( Rondón, 2011, p.89). As Anthony Giddens argues if the First Modernity is defined by romantic love, the second one it is defined by confluent love. It has also been claimed that the patriarchal family has changed to another more egalitarian and democratic one, detached from the structural parameters and transformed into a cultural form that coexists with diverse patterns of coexistence (Del Campo and Rodríguez, 2002, pp.103-133).

However as we will see below, the situation does not evolve so linearly, without forgetting that in the Second Modernity both forms of familiarized love coexisted. In this sense, in Spain today we are faced with two basic family models: one more institutional - based on romantic love - and another in which there is more choice, which is emotional and unstable and more conditioned by the satisfaction of personal needs ( Alberdi, 2005). The latter model corresponding to that of the “negotiating family” ( Meil, 2003, pp.1-16; Meil, 2006, p.11 ff.) is founded mainly on confluent love.

The first model emerged at the end of the 19th century when the connection between the concepts of romantic love, marriage, and sexuality began and is still present today. This type of love constitutes the main reason for maintaining fidelity, for the intention to marry and to form a family (Del Campo and Rodríguez Brioso, 2002, p.119) and for marital relationships more broadly, since being in love becomes the foundation for initiating and maintaining a couple ( Ferrer *et al.* 2008, pp.589-590). Thus, in 1995, 67% of the Spanish population understood that an authentic relationship lasts a lifetime, while 76% considered that true love is omnipotent, that when in true love one can cope successfully with any situation, no matter how negative it may be. On the other hand, in 2002, 70% of Spaniards agreed that romantic love can exist without marriage (Del Campo and Rodríguez Brioso, 2002, pp.119-120).

In Spain, the model of romantic love and the myths associated with it are those that prevail in our society. Indeed, this model continues to show great strength in female socialization, becoming its backbone and priority as a life project, as women turn to the private sphere, while men turn to the public sphere. For women then, the most important thing is social recognition, with love taking second place. As an example of this, in an empirical study by Ferrer *et al.* (2008, pp.589-592), it is concluded that the “Eros” style or passionate or romantic love is the most accepted by more than 80% of the people interviewed and this is true of all groups, both men and women. It is therefore evident that in in Spain there is a high valuation of romantic love ( Ubillos *et al*., 2001).

To reinforce this concept, it is worth checking the degree to which romantic myths have become established in Spain. The fact is that these myths, which are beliefs with a strong emotional charge, which concentrate many feelings and tend to create and preserve group ideology, are resistant to change and reasoning. Besides, they constitute “the set of socially shared beliefs about the supposed “true nature of love”, and, therefore, normally the romantic myths are “fictitious, absurd, deceitful, irrational and impossible to fulfill” ( Ferrer *et al.*, 2010, p.7). In a 1995 interview carried out in Spain by the Centre of Sociological Research on the presence and social acceptance of myths about love, it was found that those consulted, in general, agreed or very much agreed with these myths ( Barrón *et al.*, 1999, p.66 ff.). Specifically, “the myth of the couple” is accepted by 95% of the population interviewed; that of “fidelity” by 80%; that of “omnipotence” by 75%; that of “marriage” by 67%; that of “eternal passion” by 63%; that of “exclusivity” by 55%; that of “the better half” by 51%; and the myth of “the equivalent” is accepted by 45% of the population questioned ( Table 1).

**Table 1.** Description of myths about romantic love. Source: ( Ferrer *et al.*, 2010, p.13-14).

Myth assessed	Description
Myth of the better half	1) Somewhere there is someone predestined for each person (“your better half”).
Myth of the eternal passion	2) The intense passion of the early days of a relationship should always last.
Myth of omnipotence	3) Love is blind.
Myth of marriage	4) Marriage is the grave of love (reverse).
Matching Myth	5) It is possible to be happy without having a (reverse) relationship. 6) Separation or divorce is a failure.
Myth of jealousy ( *)	7) Jealousy is a proof of love.
Myth of ambivalence ( *)	8) You can love someone you mistreat. 9) You can mistreat someone you love. 10) True love can do everything

(*) This myth has not been analyzed in this paper.

Another empirical work ^4^ ( Ferrer *et al.*, 2010, pp.16-29) about the establishment of romantic myths in Spain shows that the acceptance of the myths of “omnipotence” (73%), “the eternal passion” (72.3%), “marriage” (71.3%) and “the better half” (52.6%) dominate ( Table 2). In this research conducted by Ferrer *et al.* (2010), the number of couples is measured through cohabitation and the acceptance of those myths of “omnipotence”, “marriage” and “pairing” and it shows the level of satisfaction with the couple’s relationship and the acceptance of these same myths. Consequently, romantic love is a very widespread experience, which is linked to the persistence of a series of clichés rooted in a traditional, romantic conception of love that helps to preserve the power structure and inequality of love relationships. It also connects that romantic vision with the majority of couples and marriages in Spain.

**Table 2.** Percentages of the number of people who believe the myths of romantic love in Spain. The total number of people interviewed is 1351 ( Ferrer *et al*., 2010, pp.11, 16).

	Disagreement	Indifference	Agreement	Do not know/Did not answer
Myth of the better half	429 (31.8%)	171 (12.7%)	711 (52.6%)	40 (2.9%)
Myth of the Eternal Passion	253 (18.8%)	78 (5.8%)	978 (72.3%)	42 (3.1%)
Mito of the omnipotent	372 (27.5%)	115 (8.5%)	820 (60.7%)	44 (3.3%)
Myth of omnipotence	230 (17.0%)	92 (6.8%)	987 (73.1%)	42 (3.1%)
Myth of marriage	963 (71.3%)	146 (10.8%)	199 (14.7%)	43 (3.2%)
Pairing myth	247 (18.3%)	135 (10.0%)	924 (68.4%)	Four. Five (3.3%)
Myth of the pairing	732 (54.2%)	126 (9.3%)	451 (33.4%)	42 (3.1%)

In a recent empirical sociological essay (Rodríguez-Santero *et al.*, 2017, pp.1-13), addressed to university students in Seville about love, it is concluded that the interviewees have an idealized and romantic concept of love in which sexuality and the more passionate or attractive aspects are secondary. Thus, in this work, the most accepted style of love, the “Agape”, the altruistic love oriented to the good of the other and of renunciation, which understands the relationship as a denial of individualism and as a process of abnegation or voluntary relinquishment of one’s desires, affections or interests for the benefit of the loved one: surrender and sacrifice and the phrase “for life” corresponds to the parameters of romantic love. Again, it should be remembered that the ideology behind this type of love expresses a form of modern patriarchy. However, in their conclusions, the authors stress that “we cannot confirm that these statements correspond absolutely to reality”, that they are more “perceptions” (Rodríguez-Santero *et al.*, 2017, p.11), that is, that they have a great imaginary component coated with a strong idealism.

Therefore, according to these empirical works, romantic love dominates in the perception of Spanish behaviors and mentalities. Therefore, I will now try to corroborate, through the analysis of two other empirical studies, how romantic love is formalized, familiarized, and institutionalized, if at all, through the different family models existing in Spain. In the 2006 study by Navarro (2006, pp.123-135), the results show that of those surveyed, 45% identify more with the ideal model of the “symmetrical family” (see Table 3): an egalitarian, democratic and tolerant model, the negotiating family, with less weight on conventions and traditional customs. Of those included in the survey, 27% were in favor of the traditional model, the “lifelong” one, rooted in romantic love. However, the “intermediate” family - which makes up 23% of those included in the study - perhaps a mixture of the traditional family and symetric family, is a type of family that could be called paradoxical, since it simultaneously combines characteristics of the other types of family without overtly opting for either of them. In any case, the symmetrical, triumphant model is ideal, since day-to-day behavior and social practices, despite progress, do not conform to this desire for equality, as we have seen in the section devoted to the characteristics of the Spanish family. As is evident, the traditional model continues to be of considerable importance.

**Table 3.** Family relationship models. Source: Own elaboration based on Navarro 2006, p.121.

Family model according to navarro		Family model according to J.A.Roche	Types of love	Percentage of interviewees who ascribed to one of the typologies
(1) “Symmetric family”	A family in which both men and women work outside the home and share the housework and childcare	Negotiating and democratic family	Confluent love	45%
(2) “Intermediate family”	A family where the woman work fewer hours outside the home and therefore take more care of household chores and childcare	The two types of family are mixed paradoxical family	Two types of love live together	23%
(3) “Traditional family”	A family where only the men work outside the home and only women take care of household tasks and childcare	Patriarchal family	Romantic love	27%

**Table 4.** A typology of Spanish families, based on the internal relations between parents and children and the parents’ finalist values ^5^. Source: Own elaboration based on Elzo 2004, p.220.

N°.	Name (according to Elzo)	Percentage of families	Author’s definition	Type of love
1°	Family, “familistic”, endogamic	23.7 %	Traditional patriarchal family	Romantic love
2°	Conflicting family	15.0 %	Dramatic family	Excessive emotional enthusiasm
3°	Nominal family	42.9 %	Cold family	Without loving enthusiasm
4°	Adaptive family	18,.4 %	Negotiating or democratic family	Confluent love
	Total parents N=1000	100.0 %		

The second work devoted to Spanish family models is especially useful to the research on this topic. It was written by Javier Elzo (2004, pp.205-229), for the *Foundation for Support for Drug Addiction,* 2002 *(FAD)* and although it focuses on parent-child relationships, it also addresses issues that can help us to infer vital information on the couple’s emotional interrelationships. Specifically, it reveals the existence of four types of families in Spain ( Table 3). In the “nominal” family, which I refer to as a “cold family” because of its non-emotional characteristic, which represents the majority of families in Spanish society (42.9%), the parent-child relationship is one of peaceful co-existence rather than one of participative cohabitation. Moreover, family members communicate little and do not participate in common objectives, as they have decided to do in order not to create conflicts. The “adaptive” family model (18.4% of those surveyed) is the most recent and the most modern (Second Modernity) and is the model that best reflects family tensions. This is the negotiating family I referred to earlier, as it is the one with good communication and continuous role review. However, there is a strong risk of ruptures due to misunderstandings within the couple and between the couple and the children. The most traditional model within this is that of the “family and familistic and endogamic” family (23.7% of those surveyed), in which the parents have strong identities. Furthermore, this model is closed and such families are unconcerned with the problems of the world, although good relations and a warm home enviroment full of affection among family members do also exist within this model. Finally, the “conflictive” family model (15% of those surveyed), which I call a “dramatic family”, is the one in which there is the most conflict (mainly due to drugs and sexual issues, relations between siblings, and between parents and children).

Javier Elzo does not quote the term “romantic” to refer to the four models and, indeed, it does not seem appropriate to define them as such, except for one of them: the “family familistic and endogamic”, the second largest, whose characteristics resemble some elements of romantic love. The “adaptive” family (the third most extensive model) does clearly refer to the negotiating family, while the majority family, the “nominal” one, is not defined by its enthusiasm or love intensity, nor is this the case in the less established model, the “conflictive” family.

## Conclusions

In this article, I have tried to confirm how the two types of modern love are institutionalized, formalized, or familiarized, firstly by putting them into the context of the Spanish family structure and, secondly, by revealing how the two coincide, as well as denoting which is the primary and which is the secondary form. In this respect, the Spanish care model and family structure tend towards individualism, but not without resistance from the old patriarchal system. Thus, the analysis of numerous works, both theoretical and empirical, has allowed me to confirm the five great transformations of the Spanish family structure: progressive reduction and de-institutionalization, heterogeneity, privatization, negotiating capacity, and a plurality of forms. All of these transformations demonstrate that the Spanish family is indeed immersed in the general European context of individualization but with unique nuances and markedly ambivalent characteristics since the Spanish family is situated halfway between the traditional and the modern, the communal and the individual.

The same goes for love, which is also subject to the same process of reduction and individuation. Concerning the two types of love in Spanish families, four final considerations have been inferred:

1) It has been revealed that likely the most institutional love - the “familistic and endogamic family”, based on romantic love - and the prevalence of the myths associated with it are still common in Spain, according to five of the six studies. If these studies are correct, this love would manifest in a series of clichés rooted in a traditional romantic conception of love that contributes to preserving the power structure and the inequality of love relationships. Alongside romantic love, there is the majority form according to Ferrer *et al.* (2008) empirical work s. This form corresponds to the “negotiating family”, “adaptive” or “symmetrical” model, and is mainly based on confluent love. Finally, there are two other institutional forms of love: the “nominal” and the “conflictive” family ( Elzo, 2004).
2) However, as those researchers who were consulted recognize, although the desire for romantic love is widely established and is the majority in Spanish society, it is more of an ideal, more of a perception than a practice. This is also the case with the “symmetrical” or negotiating family and confluent love, which expresses an ideal of equality not yet achieved in social practice. In the case of “nominal” and “conflictive” love, they do not seem to exhibit any idealization, but on the contrary, these depict a crude reality in which, although the family is sustained, it does so without the affective quality it would require, since either communication is annulled in the first case, or it is dramatized and problematized, in the second case.
3) The familiarization of the forms of love in Spanish society (love is, by nature, ambiguous) are therefore determined by ambivalence, as the two basic types of love - the romantic and the confluent - coexist. With them also coexists two ideological conceptions of love, patriarchal and democratic; the first being the most accepted by Spanish society today, while the second continues to develop in terms of familiarity and popularity.
4) This contradicts the evolutionary linearity expressed by some specialists who understand that the Spanish family has gone from being romantic in the First Modernity, to confluent in the second. However - as I have noted here - in the Second Modernity, both modern types of love and familiarity coexist, along with two others: the “cold family” and the “dramatic family”. Added to this is the contradiction that, although the evolution of the family structure places the Spanish family within the Second Modernity, the same does not seem to have happened with affective interrelationships, more typical of the First Modernity; that is, it is a legacy of the mentality and family structure of Spanish patriarchal culture. This means that - in matters of love - the old is not completely over and the present has not been definitively installed, that the present and the past coexist paradoxically without there being a clear differentiation between the two and neither becomes the dominant paradigm. Thereby, this is reinforced by the existence of the other two models, hybrid and undefined, and of poor emotional quality, which - if Elzo is right - could represent, as a whole, most of Spanish society. Nonetheless, they denote the deep disorientation and crisis in which family affairs find themselves today.

In conclusion, although most analysts believe in the strength of the nuclear family and the positive consideration of marriage in the Mediterranean and in Spain in particular, something is wrong. The fact is that a careful, contrasted analysis of the theoretical and empirical works consulted here has revealed nuances, ambivalences, and contradictions in Spanish society with regard to love and the ways in which it is familiarized. Thus, the Spanish family as a whole could be called “paradoxical”. Indeed, its reduction to the minimum expression of itself, its fragmentation and its emptying of meaning may lead one to think that, although the family institution resists this potential collapse, it is so weakened that it does not seem to have a great future. If I am right, then it would be worth reflecting on the future of the emotional ties of the spouses on which the current Spanish family is based. Therefore, the weakness of the institution implies - as has been proven - the fragility of the emotional interrelations - of love - that sustain it, since the intensity, quality, and consistency of the relations between its members and, in particular those between the spouses, have been affected.

## Data Availability

No data are associated with this article.

## Footnotes

^1^
In 2015, there were 1,897.5 thousand single-parent households in Spain. In 2020 the number of thousands of single parent families has risen to 1,944.8.

^2^
In 2020, of the 1,944.8 thousand single-parent families in Spain, 672.5 were from Madrid, and 586.9 from Barcelona.

^3^
Of the total of 1944.8 thousand single-parent households in Spain in 2020, 1582.1 were women.

^4^
In this work, the sample was selected from the general Spanish population through a sampling tool stratified by gender and, within each stratum, by quotas based on age. In total, 1,351 people were selected.

^5^
The end values are the goals you have in life, while the instrumental values are the ones you use in your day-to-day life to reach the end values.
