# Supplementary material for: The paradoxes of love in the Spanish family: a sociological approach
Source: F1000Res. 2021 Jul 21;10:627. [Version 1] doi: 10.12688/f1000research.51358.1 (PMC8361805; doi:10.12688/f1000research.51358.1)
Supplement: Peer review history

# Peer review: Reviewer response for version 1

**DOI:** 10.5256/f1000research.54518.r90114

**Published:** 2021-08-31

**Authors:** Olga Alejandra Sabido Ramos

**Affiliations:** 1 Universidad Autónoma Metropolitana, Mexico City, Mexico

The article “The paradoxes of love in the Spanish family: a sociological approach”  presents a broad theoretical and empirical panorama to demonstrate the coexistence of two types of modern love (romantic and confluent) and the nuances around the notion of Spanish family based on “Mediterranean model.” According to the evidence, the Spanish family structure and love tend towards individualism but are in tension with traditional family forms. In conclusion, the romantic relationships in the Spanish family are more paradoxical and hybrid. The article has a rigorous critical apparatus and complete research of current literature. Likewise, it stands out for its sophisticated analytical and interpretive construction. This analytical horizon allows us to introduce nuances in the ideal types about family and love. This input is an excellent contribution to the research of these topics.

Is the work clearly and accurately presented and does it cite the current literature?

Yes

If applicable, is the statistical analysis and its interpretation appropriate?

Yes

Are all the source data underlying the results available to ensure full reproducibility?

Yes

Is the study design appropriate and is the work technically sound?

Yes

Are the conclusions drawn adequately supported by the results?

Yes

Are sufficient details of methods and analysis provided to allow replication by others?

Yes

Reviewer Expertise:

Sociology of emotions, Embodiment and sensory studies, Sociological Theory.

I confirm that I have read this submission and believe that I have an appropriate level of expertise to confirm that it is of an acceptable scientific standard.

# Peer review: Reviewer response for version 1

**DOI:** 10.5256/f1000research.54518.r90115

**Published:** 2021-08-17

**Authors:** Elaine da Silveira Leite

**Affiliations:** 1 Department of Sociology and Politics, Universidade Federal de Pelotas, Pelotas, Brazil

The article presents theoretical-methodological consistency and quality, and it also cited the current literature. The research evidence is the result of a review of the literature. The author demonstrates that the Spanish family and love are immersed in the general European context of individualization, but distinctly characteristics, in which the Spanish family is situated halfway between the traditional and the modern, the communal and the individual. The article presents a clear and objective writing.

Is the work clearly and accurately presented and does it cite the current literature?

Yes

If applicable, is the statistical analysis and its interpretation appropriate?

Not applicable

Are all the source data underlying the results available to ensure full reproducibility?

Yes

Is the study design appropriate and is the work technically sound?

Yes

Are the conclusions drawn adequately supported by the results?

Yes

Are sufficient details of methods and analysis provided to allow replication by others?

Yes

Reviewer Expertise:

Sociology, family, budget, emotional ties, intimicy.

I confirm that I have read this submission and believe that I have an appropriate level of expertise to confirm that it is of an acceptable scientific standard.

# Peer review: Reviewer response for version 1

**DOI:** 10.5256/f1000research.54518.r90112

**Published:** 2021-08-12

**Authors:** Jose Miguel Beriain Razquin

**Affiliations:** 1 I-Communitas, Institute for Advanced Social Research, Public University of Navarre, Navarre, Spain

The paper is an excellent contribution to the sociology of the family highlighting the features of the "Spanish family" closer in its shape, and challenges to the families of the modern complex societies. The family in Spain has its own "Mediterranean" features but at the same time faces the challenges, beyond any communitarian background, of a fast process of modernization within the fields of market economy and the modern lifestyles.

 Family in Spain is a symbolic substitute of the lacks of religion which has been quickly privatized and a functional substitute of the market which is not sufficiently dynamic in order to absorb huge amounts of labor force.

 To my account the paper can be indexed in his present version without any change.

Is the work clearly and accurately presented and does it cite the current literature?

Yes

If applicable, is the statistical analysis and its interpretation appropriate?

Not applicable

Are all the source data underlying the results available to ensure full reproducibility?

Yes

Is the study design appropriate and is the work technically sound?

Yes

Are the conclusions drawn adequately supported by the results?

Yes

Are sufficient details of methods and analysis provided to allow replication by others?

Yes

Reviewer Expertise:

Sociological Theory, Sociology of Religion and Cultural Sociology

I confirm that I have read this submission and believe that I have an appropriate level of expertise to confirm that it is of an acceptable scientific standard.

# Peer review: Reviewer response for version 1

**DOI:** 10.5256/f1000research.54518.r90111

**Published:** 2021-08-12

**Authors:** Adrián Scribano

**Affiliations:** 1 National Scientific and Technical Research Council (CONICET), Buenos Aires, Argentina

The article refers to the connections between the different modalities of love and the types of families associated with them and pointing out their impact on the Spanish social structuring process. In this context, the author intends to: characterize and define the basic features of the "Spanish family" in relation to the society of individualization; investigate the forms of institutionalization of modern forms of love, with special emphasis on those known as romantic love and confluent love; discuss if different types of love coexist and how this it's possible and if there is some kind of pre-eminence among them and analyze the strength / fragility of the family institution in relation to the affectivity that sustains them.

 The author shows in the first section the conceptualization of the practices called romantic love and confluent love as modern modalities of instantiation of love as tensions between different ways of managing the formal / informal, proximity / distance, dogmatisms / flexibility that involves these various ways of affectivity. In this context, the author declares their special interest in love as a fundamental component of the family and the conjugal couple.

 The section that follows involves the discussion of "the Spanish family" in connection with the individualistic family model of northern Europe, where it begins by presenting how the forms of romantic and confluent love are institutionalized in Spain through their familiarization. Said section ends by stating:

*“In sum, this transformation of the state of the family is likely to manifest not as an unequivocal trend towards individualization, but rather a reformulation of the concept of family in terms of roles, functions, and face-to-face relations with other institutions”* (page 7).

 Then the article is dedicated to establishing the five structural characteristics of the Spanish family: progressive reduction and deinstitutionalization; heterogeneity; privatization; the modification of negotiating capacity; and the plurality of forms. It ends by holding that these five great transformations of the Spanish family have broken with the traditional system where interculturality and gender equality synthesize the most important modifications for the social system.

 In the context of their argument, the author proposes three types of family modalities in connection with forms of love: a) Negotiating and democratic family with Confluent love; b) The two types of family are mixed paradoxical family with two types of love life together and c) Patriarchal family and Romantic love.

 The article concludes that the state of the Spanish family as a whole could be called "paradoxical", provoking to think that it would be worth reflecting on the future of the emotional ties of the spouses, accepting that the weakness of the institution implies the fragility of the interrelations emotional - of love - that sustain it, since the intensity, quality and consistency of the relationships between its members and, in particular, between spouses, have been affected.

 The article offers an adequate review of the literature and an organization of the theoretical instruments in connection with the objectives of the writing, in this sense, perhaps a greater approach to the works from other disciplinary fields of sociology and anthropology could have been included, but this does not affect the development of the argument.

 The work presents an argumentative structure that goes from conceptualization, through the systematization of empirical information until it reaches a creative hermeneutic of the connection between family types and forms of love where the originality of the author's proposal can be observed.

 The statistical data consigned are identifiable and easily accessible so that every reader is in a position to validate both its presentation and meaning, in this direction it can be said that in the context of a necessary plural framework of analysis the conclusions are appropriate.

 In the context of the above, I believe that a way of understanding even more what the author argues in connection with the proximity / distance of the Spanish family organization, the individualization processes and the romantic and / or confluent modalities is possible to be captured by through qualitative information coming from the current “youth popular culture” in Spain:

1. The 'influencer' Naim Darrechi, who boasts of not using a condom and deceiving girls saying that he is sterile, has 26 million followers on TikTok and adds up to more than seven million followers on Instagram, which as is known are the most popular applications among young people.
2. Farruko has more than 32,000,000 monthly listeners on Spotify and his hit, Pepas, had in June more than 64,000,000 reproductions where it is said repeatedly:
  *"I do not care what they say about me*
  *Life you your life, that I live Mine*
  *That is only one, enjoy the moment*
  *That time is running out and does not go back*
  Drinking, smoking and f***ing"
3. If you search Google Trends for the words love, sex and family between 2016 and 2021 in Spain you will find that the following order of importance love, sex and family and within love the most sought after is the Turkish series "I rent my love to you" that Google describes as follows:
  *"Six months is the time a young woman will have to fall in love, marry and leave a successful businessman. The protagonist, desperate to pay off a family debt, will accept the surprising challenge of love proposed by the aunt of the millionaire designer."*

These simple brushstrokes from popular culture bring us closer to those that the author of the evaluated article indicates as a territory to explore the modalities of a politics of sensibilities in a normalized society in immediate enjoyment through consumption where the politics of perversion, the banalization of the good and the logic of waste are presented as its central axes.

Is the work clearly and accurately presented and does it cite the current literature?

Yes

If applicable, is the statistical analysis and its interpretation appropriate?

Yes

Are all the source data underlying the results available to ensure full reproducibility?

Yes

Is the study design appropriate and is the work technically sound?

Yes

Are the conclusions drawn adequately supported by the results?

Yes

Are sufficient details of methods and analysis provided to allow replication by others?

Yes

Reviewer Expertise:

Sociology of Emotions, Sociology of Body, Social Theory

I confirm that I have read this submission and believe that I have an appropriate level of expertise to confirm that it is of an acceptable scientific standard.
